# Introducing *Alphitobius diaperinus*, (Insecta: Tenebrionidae) as a New Intermediate Host of *Hadjelia truncata* (Nematoda)

**Published:** 2012

**Authors:** AR Alborzi, A Rahbar

**Affiliations:** 1 Department of Pathobiology, Faculty of Veterinary Medicine, Shahid Chamran University of Ahvaz, Ahvaz, Iran; 2 Dept. of Parasitology, Faculty of Veterinary Medicine, Shahid Chamran University of Ahvaz, Ahvaz, Iran

**Keywords:** Pigeon, *Alphitobius diaperinus*, *Hadjelia truncata*, Intermediate host

## Abstract

**Background:**

*Hadjelia truncata* is a nematode that causes lesions in the gizzard lining of pigeons, which may even lead to death. The aim of this study was to introduce *Alphitobius diaperinus* as a new intermediate host for *Hadjelia truncata*.

**Methods:**

*H. truncata* infection was identified in a pigeon flock in Ahvaz City, Khuzestan Province, Iran by performing fecal examination and autopsy. Adult and larval stages of beetles were collected from the litter of pigeon houses, and identified morphologically. The beetle larvae were cultured in a medium, containing feces of the infected pigeons. Nematode larval stages from naturally and experimentally (culturally) infected adult beetles were fed to two groups of pigeons

**Results:**

The collected beetles were identified as *Alphitobius diaperinus*. Average length and width of the adult beetles were 6.31 mm and 2.88 mm respectively. Infection rates of naturally and experimentally infected beetles with larval stages of the nematode were 66.2% and 45.1% respectively. The adult nematodes collected from gizzards of experimentally infected pigeons were identified as *H. truncata*. Nematode infection rates in pigeons after feeding the infective larvae collected from naturally and experimentally infected beetles were 44.7% and 32.5% respectively.

**Conclusion:**

*A. diaperinus* can serve as a natural intermediate host for *H. truncata*.

## Introduction

*Hadjelia truncata* (Creplin, 1825) is a nematode from the family Habronematidae that lives in the gizzard of a number of birds in Europe and Asia; including pigeon (*Columbia livia domestica*) and hoopoe (*Upupa epops*) (1–3). It causes lesions in the gizzard lining of pigeons, which may even lead to death. It has an indirect life cycle and some species of insects has been reported to serve as intermediate hosts; in the hemocoel of which the infectious stage (L3) develops. Chabaud reported development of the nematode's larval stages in the haemocoel of the beetles, *Asida jurinei*, *A. sericea* and *Phylan abbreviates* (3, 4). The lesser mealworms, *Alphitobius diaperinus* (Panzer) (Coleoptera: Tenebrionidae), also known as the darkling beetles or litter beetles are common cosmopolitan pests in poultry houses (5, 6, 7). Both adults and larvae are scavengers (8), consuming chicken food, feces, and dead birds, and sometimes attacking live birds (9, 10). Darkling beetles are important vectors of a number of poultry pathogenic agents. These include some viruses, bacteria, fungi, and protozoa (11–18). The beetle can serve as an intermediate host for helminths such as fowl cestode, *Choanotaenia infundibulum*, other chicken tapeworms (19), and the nematode, *Subulura brumpti* (20). Culturing lesser mealworms in the laboratory is needed to produce adult beetle for experimental studies. Different culture methods have been described earlier (21, 22).

This study was performed in order to distinguish the cause of mortality in a pigeon flock in Ahvaz City, center of Khuzestan Province, Iran. Furthermore, to our knowledge, the beetle has not yet been determined as an intermediate host for *H. truncata* under experimental conditions.

## Materials and Methods

### Case history

There are several pigeon breeders with small and large flocks in and around Ahvaz City. The flocks consist of homer, fancy or mixed pigeons and sometimes are seen as roller pigeons especially in the sky of rural regions. Clinical signs of *H. truncata* infection include decreased food intake, tendency to eat soft and wet foods, regurgitation of semi-digested food and decrease in sexual libido in both sexes and in egg laying. Weakness and mortality were also observed in a number of pigeons in a large flock.

Fecal samples from the pigeons of the flock were collected separately, and examined microscopically with the use of direct and Clayton-lane fecal floatation techniques. In postmortem examination of two dead pigeons, the gizzards were enlarged and full of undigested material, the intestines were almost empty and a large number of pink-red worms were found in the region between their proventriculus and gizzard, underneath the mucosal layer of the isthmus of proventriculus and mainly beneath the lining of gizzards especially anterior part of the soft koilin layer. The worms were collected from the gizzards, counted, and maintained in 70%-ethanol-5% glycerin. Some of them were cleared with lactophenol for identification.

### Collection and Culturing beetles

After collecting the bedding litter (fecal layers, soil, piles and broken feathers) from the pigeon houses, they were placed into plastic bags, transported to the laboratory and examined under a stereomicroscope. Numerous adults and larval stages of the beetles were observed in litter samples. The larvae and adult beetles were collected from the litter samples by sieving them with common sieves.

The beetles were cultured with the modified method of Rice et al 2009 (21). The beetle larvae were put into a plastic box (measuring 135×195×80 mm) that contained 250 ml of culture medium (up to 15% sieved dust of litter, 15% wheat bran, 70% layer and semi-moist fecal pellets from cage^'^s tray of pigeons infected with the nematode) and a number of washed apple halves placed faced down on the medium. The plastic boxes were sealed with gauze and lid of the box, which closed the vent while allowing passage of air, and maintained for up to 5 months under 27-34°C temperature and relatively high humidity.

### Identification of beetles and their nematode larval infection

Collected beetles were identified morphologically and the mean length as well as width of 70 beetles were measured. To confirm natural infection of the beetles and evaluation of the possibility of experimental infection, 133 adult beetles from litter samples (natural group, A) and 51 from cultured larvae (experimental group, B) were killed and their exoskeletons removed under stereomicroscope by means of forceps and dissection needle. Their haemocoel in the abdomen, thorax, and head regions were examined for the presence of the nematode larval forms. Collected free larvae or cyst forms of each beetle were counted and active forms (third stages) of them were used for further experiments. Third stage larvae were also measured by micrometer.

### Preparing pigeons and experimental infection

Seven pigeons, free of any nematode infection in their alimentary tracts, were kept in separate cages. They were divided in 2 groups: P1 (n = 5), P2 (n = 2) and each bird was fed 40-50 nematode larvae, which were collected from naturally (P1), and experimentally (P2) infected beetles. In the second month post infection, their feces were examined daily. In addition, the infection rate of larvae fed to pigeons were determined by collecting and counting the larvae from the alimentary tract, especially gizzard, proventriculus and the region between them in two pigeons which died in days 9 and 18 after infection; and also by counting the collected nematode adults from the remained pigeons until 4 months after infection.

## Results

**Fig. 1:**
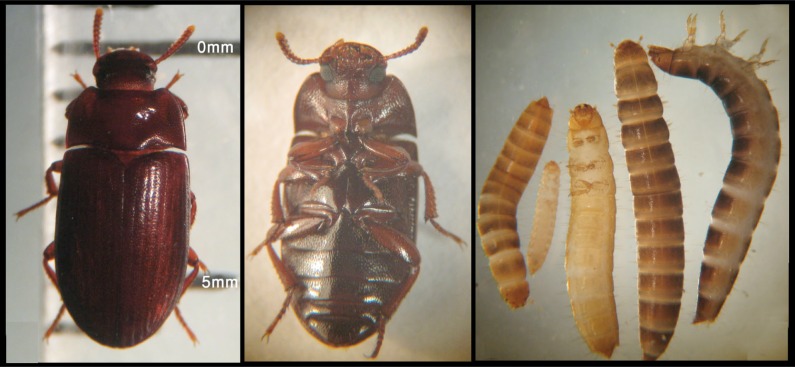
Dorsal, ventral surfaces of adult (left) and some larval stages (right) of *Alphitobius diaperinus* collected from the pigeon houses in Ahvaz City, Khuzestan Province, Iran

**Fig. 2:**
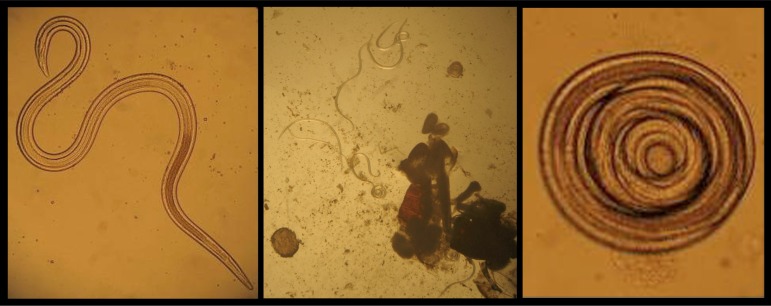
Free third-stage (left) and cystic form (right) larvae of *Hadjelia truncata* collected from hemoceol of experimentally infected the beetle, *Alphitobius diaperinus* (middle). The pictures were taken by a digital camera in the Parasitology Lab of the Faculty of Veterinary Medicine, Shahid Chamran University of Ahvaz

All of the collected beetles from the pigeon house were identified as *Alphitobius diaperinus* (Fig. 1), due to the characteristics of adults, described by Dunford et al. 2004, 2009. Adult beetles are broadly oval, moderately convex, black or brownish-black in color and usually shiny in appearance. Color can be variable depending on the age and size. Antennae are densely clothed with short yellowish hairs, with the terminal segment lighter in color. The head is deeply emarginated in front, has a distinct clypeal groove, and the surface is coarsely punctured. Eyes are emarginated approximately one-half the length of eye, and prosternal process is horizontal between forecoxae (23, 24). Average length and width of the collected adult beetles were 6.31 mm (5.54 to 6.96mm) and 2.88 mm (2.50 to 3.20 mm) respectively. The number and percentage of infected adult beetles, their nematode burden and type of larvae (free or cystic forms) (Fig. 2) are summarized in Table 1.

**Table 1:** Infection of *Alphitobius diaperinus* (beetle) adults with larval stages of *Hadjelia truncate*

Group- No. of beetles	No. infected beetles (%)	No. infected with cyst form (%)	No. infected with free larvae (%)	No. infected to cyst form + free larvae (%)	Mean No. of cysts in infected beetles	Mean No. free larvae in infected. beetles
**A-131**	88 (66.2)	40 (45.5)	80 (90.9)	(36.4) 32	6.7	4.4
**B-51**	23(45.1)	10 (19.6)	15(29.4)	10(19.6)	6.2	2.7

Average length of the nematode third-stage larvae were 4.44 mm (range 3.8 to 4.95 mm). Furthermore, 3 out of 51 (5.9%) beetle larvae were infected with larvae and mostly cysts, with the mean of 1.6 larvae (ranging 1 to 3) per each larva. The maximum prepatent period recorded in experimentally infected pigeons was 50 days. Infection rates of pigeons are summarized in Table 2. The percentages of the male and female adult nematodes collected from the pigeons were 38% and 62% (with the approximate proportion of 1 to 2) respectively. The nematode eggs were oval and light green, measuring 42×27µm and containing embryos.

**Table 2:** Infection rate of *Hadjelia truncata* in pigeons infected with the larvae of *Alphitobius diaperinus*

Groups	No. given larvae	Time of necropsy (days post infection)	No. collected			Total parasite burden	Infection rate (%)
			Adults		larvae		
			M	F			
P1-1	40	9	0	0	16	16	40
P1-2	50	18	0	0	18	18	36
P1-3	40	120	14	7	0	21	52.5
P1-4	40	120	13	5	0	18	45
P1-5	40	108	10	10	0	20	50
**Mean No**.	–	–	12.3	7.3	17	18.4	44.7
P2-1	40	120	8	4	0	12	30
P2-2	40	120	8	6	0	14	35
**Mean No**.	40	120	8	5	0	13	32.5

## Discussion

*Hadjelia truncata* has been reported from the alimentary tract of a variety of bird species in Europe and Asia. There have been several reports of *H. truncata* infection in pigeons from Iran (25), Egypt (26, 27), Iraq (28), and Cyprus (29). Although *H. truncata* is rarely reported as pathogenic, but in all of the reports, clinical signs, or pathological effects were described. The nematode has been formerly reported in gizzards of pigeons from Ahvaz; but without any marked clinical signs (28). However, clinical signs and gross pathological changes were seen in the present study; especially, the enlargement of gizzard of the naturally infected birds which did not happen in those experimentally infected. Therefore, it was indicated that clinical disease is depended on the number of worms.

Results of the study showed that adult lesser mealworm *(Alphitobius diaperinus)* is a natural intermediate host for the nematode *H. truncata*, and indicated the potential of the nematode to infect experimentally of the beetles. The only investigation on natural infection of *A. diaperinus* to *H. truncata* encysted larvae was carried out by Geyhan, M. E (2004) in Assiut Governorate of Egypt; which as far as we know is not published (30). In this work, 19.91% of beetles (1170 beetles were dissected) were naturally infected with the encysted larvae. Natural infection rate of the beetles with the nematode larvae in our study was higher in comparison to the mentioned study. The reason of this difference could be mainly higher infection intensity of the pigeons to the adult nematodes. Overall, both researches especially the experimental infection confirmed natural potential of the beetles to act as an intermediate host for the nematode *H. truncata*.

Chabaud (1951, 1954) reported the development of the nematode eggs to third stage larvae in the haemocoel of other beetles such as Asida jurinei, A. sericea and Phylan. At about 18-20 °C the first moult took place in the insect in 25 days and the second in 50 days. Growth was most marked after 35 days (from 1.8 to 4.0 mm). In our study, collected third stage larvae were morphologically similar to what described by Chabaud; as they had large pseudolabia and a divided esophagus and that their tail ended in a peculiar unarmed button-like appendage; but the average length of larvae was more than what had been achieved by Chabaud. Comparison between the two results indicates that growth of the larvae in the beetles continues even after 35-50 days.

Infection of the beetle larvae with larval stages of the nematode indicate that they may have a role in the development of the nematode; but not as important as adult forms. Because the infection rates of beetle larvae were very low (5.9%, ranging 1 to 3 cysts in each), in comparison to the adults (66.2). It is not clear if the infective stage of the nematode can be produced in larvae of the beetles. Karunamoorthy et al. (1994) have described *A. diaperinus* as an intermediate host for the nematode *Subulura brumpti*, a common parasite of the cecum of poultry in many parts of the world (2).

As the lesser mealworm, *A. diaperinus* is an important vector or reservoir of many avian parasites and pathogens, including: some cestodes (19), protozoa, (16, 31), viruses of (3, 16, 17, 32), some bacteria. (11, 18, 33–35) and fungi (13, 36); transmission of the parasites and pathogens by the beetle to pigeons is probably of great importance, especially when large flocks of pigeons live near poultry houses.

Pigeons eat grains, seeds and occasionally insects especially in their brooding situation, therefore this may be a cause of high infection rates in the housed pigeons. It seems that starvation and unavailability of proper food (grains, seeds) forces them to eat the infected beetles in the bird droppings. Infection rate in naturally infected beetles with nematode larvae were more than those experimentally infected; which is probably because of the adult beetle life span and high probability of repeated infection, as the adult beetles are long lived and normally persisting for more than a year (19). It appears to be important in potential long time transmission of pathogenic agents. In addition, climatic condition of the region (Khuzestan) is relatively suitable for growth of the beetles; since the lesser mealworm is well suited for warm and humid conditions. This situation also exists in poultry or brooder houses (6). However, there is no published report of the beetle in pigeon or poultry houses in Ahvaz and not in Iran. The beetle has been reported in pigeon houses in the Sudan (37). Development of the nematode larvae in the beetles does not have apparent and serious effects on them. The length range of the beetles was slightly greater than those of reported by other investigators (24); which was possibly related to geographical differences.

In conclusion, the study revealed that the lesser mealworm (*A. diaperinus*) is a natural intermediate host for *H. truncata* and development of the nematode from fed eggs to infective stage (third- stages) takes place in haemocoel of the beetles.
